# Community Collaborations & Technology Successfully Connect Isolated Older Adults to Education & Activities

**DOI:** 10.1093/geroni/igab046.3342

**Published:** 2021-12-17

**Authors:** Laura Spradley

**Affiliations:** UAMS Arkansas Geriatric Education Collaborative (AGEC), Little Rock, Arkansas, United States

## Abstract

Providing programs, activities and education to older adults (OA) is a challenge under normal circumstances. The Arkansas Geriatric Education Collaborative (AGEC) is a HRSA Geriatric Workforce Enhancement Program with a mission to “provide high quality programs that support healthy aging in Arkansas.” Prior to the pandemic, AGEC educators provided face-to-face programs to OA’s through senior centers, places of worship and other public venues. The pandemic changed all that. In-person programs were replaced with zoom presentations, social media events and pre-recorded programs placed on websites and patient-learn platforms for 24/7 viewing. Gaining viewership proved difficult and after collaborative research, it was determined the major barrier was a digital divide between access, usage and knowledge of digital platforms. To overcome this barrier, AGEC utilized TV, radio, libraries, digital infographics, newsletters and video tips addressing Wi-Fi and technology training. Videos, distributed via multiple routes, addressed basic topics such as “Creating and Utilizing Zoom and Facebook accounts” and “how to improve telehealth visits”. After establishment of a regular audience, AGEC engaged new and established partners and hosted a plethora of educational programs and activities further expanding the viewing audiences. In addition, with personalized emails and targeted marketing, AGEC engaged OA audiences in caregiving workshops, on-line caregiver support groups, telephone check-ins and exercise programs. Many OAs have found ways to bridge the digital divide and are engaged and active with educational and program activities and have used their new skills to connect with other OAs, grandkids, friends and even their spiritual communities.

